# Extraskeletal myxoid chondrosarcoma of the vulva: A case report

**DOI:** 10.3892/ol.2015.3586

**Published:** 2015-08-11

**Authors:** ALISA VILLERT, LARISA KOLOMIETS, NIKOLAY VASILYEV, VLADIMIR PERELMUTER, OLGA SAVENKOVA

**Affiliations:** 1 Department of Oncogynecology, Cancer Research Institute, Siberian Branch of the Russian Academy of Medical Sciences, Tomsk, Russia; 2 Department of Clinical Pathology and Cytology, Cancer Research Institute, Siberian Branch of the Russian Academy of Medical Sciences, Tomsk, Russia

**Keywords:** vulvar cancer, extraskeletal myxoid chondrosarcoma, diagnostics, treatment, prognostic factors

## Abstract

Extraskeletal myxoid chondrosarcoma (ESMC) of the vulva is an extremely rare tumor and currently, there is little available information on its biological behavior and treatment strategy. The present study reports a case of recurrent ESMC of the vulva in a 32-year-old female. The patient presented with an increasingly painful mass of the right vulva, at the site of an exision which had been performed 7-months previously. The tumor mass was histopathologically diagnosed as primary ESMC of the vulva and subsequently, vulvectomy was performed. Cytological examination showed negative surgical margins. Intraoperative radiation therapy at a single dose of 10 Gy was administered to the bed of the removed tumor. The patient refused chemotherapy and five months after surgery, a new lesion was identified in the inguinal region. Bilateral inguinal-femoral lymph node dissection was performed and external beam radiation therapy at a dose of 40 Gy was administered to the inguinal region. Follow-up examination seven months after surgery revealed no evidence of disease progression and at present, the patient remains alive. This study highlights the importance of analyzing each clinical case of ESMC as this may lead to the development of guidelines for the optimal treatment of this rare tumor.

## Introduction

Approximately 90% of all malignant tumors of the vulva are squamous cell carcinomas. Sarcomas of the vulva constitute a variety of malignant neoplasms that account for 1–2% of vulvar cancers (1,2); the most common are rhabdomyosarcoma, leimyosarcoma, epithelioid sarcoma, alveolar sarcoma of the soft tissues and liposarcoma (3).

In accordance with the 2013 World Health Organization (WHO) histological classification of tumors (4), sarcomas are classified into soft-tissue tumors and subclassified into malignant soft-tissue tumors. Extraskeletal myxoid chondrosarcoma (ESMC) represent <3% of all soft-tissue sarcomas (5).

Stout and Verner first described ESMC in 1953 (6). In 1994, ESMC was included into the group of soft-tissue tumors with uncertain differentiation (7).

ESMC has a male to female ratio of 2:1, with the peak occurrence in the sixth decade of life (8). ESMC is more commonly observed in the lower extremities, however, cases of ESMC of the orbit, shoulder and upper extremities have been reported (7,9,10–17). The majority of these tumors are solitary, deeply-seated, superficial, slowly-growing nodules, measuring 5–10 cm in diameter. The lesions are typically well-circumscribed and have a gelatinous appearance, often with hemorrhagic foci (18).

Light microscopic examination reveals the presence of rather monomeric, small, round or oval cells with centrally located hyperchromic or vesicular nucleus, with evenly distributed chromatin, scant eosinophilic cytoplasm and often no evident nucleolus. Cells form straight or bent linear chains anastomosing between each other and forming a lacy arcade pattern. Perivascular condensation of the tumor cells, with formation of pseudorosettes, also occurs. Tumor structures are located in the prominent basophilic myxoid slightly vascular matrix. The tumor is divided into small lobules by narrow fibrous septa of different thicknesses. The peripheral region of the tumor is characterized by high cellularity, often with the evidence of rhabdoid and epithelioid cells. Cysts, hemorrhages and geographic necroses can occur in the tumor tissues (7,19–23).

Irrespective of ESMC localization, pre-operative diagnosis may be difficult, and thus verification is carried out based on the results of core-biopsy, followed by the evaluation of immunohistochemical and histological changes in the tumor tissue (24).

In accordance with the minimum clinical recommendations of the European Society of Medical Oncology (2014) (25), wide excision of the tumor with negative margins (R0 resection) is the standard surgical method for soft-tissue sarcomas. The treatment of patients with localized ESMC should include primary tumor excision with a wide surgical margin. High-dose radiation therapy has previously been reported to elicit a response from ESMC, while adjuvant chemotherapy has been reported to elicit only a poor response (26). Radiation therapy is recommended for high-grade sarcomas, moderately-differentiated tumors, positive surgical margins and recurrent sarcoma (27–29). The two drugs with the highest established response rates in soft-tissue sarcoma are doxorubicin and ifosfamide, and this drug combination has been used as the gold standard for a number of years. However, this chemotherapy schedule has been found to be ineffective for ESMC (30). Interferon α-2b has been investigated in several studies, showing promising results (31). Gemcitabine, methotrexate and imatinib can also be administered for this type of tumor in an adjuvant regimen (32). In 2010, a study by Geyer and Karlin reported that there were no studies showing the efficiency of the modern chemotherapy regimens for ESMC (33). Adjuvant radiation therapy for ESMC is used prior to chemotherapy in cases with positive surgical margins and in cases where it is impossible to perform a resection (32).

ESMC has been described as slowly growing and late to metastasize. Due to the rarity, protracted clinical course and prolonged survival of patients with ESMC, long-term follow-up is recommended for the early detection of local recurrence and distant metastases. ESMC has been reported to have a relatively good prognosis. However, ESMC has a high potential for metastasis, particularly to the lungs, regional lymph nodes and bones (21,34–36). Enzinger and Shiraki considered that neither the tumor localization nor the tumor size affected the disease prognosis, however, the study underlined the fact that the prognosis is associated with the histological grade (9). A more recent study by Oliviera *et al* defined high cellularity, large tumor size, presence of anaplasia or rhabdoid features, high mitotic activity (>2 per 10 high-power fields) and proliferative activity (Ki-67, ≥10%; Ki-67 ‘hot spot’, ≥25%) as adverse prognostic factors (37). Meis-Kindblom *et al* analyzed 117 cases with ESMC and showed that the clinical parameters, but not the histological features, were associated with decreased survival (35).

The overall 5-, 10-, and 15-year survival rates of patients with ESMC are reported to be 82–90, 63–70 and 58%, respectively. Two-thirds of patients develop recurrences and more than half of the patients develop metastases. Local recurrences are observed in 48% of cases (half of these are multiple local recurrences) and metastatic recurrences are observed in 46% of cases (26,32,35).

The present study reports a case of recurrent ESMC of the vulva in a 32-year-old female. Written informed consent was obtained from the patient.

## Case report

In June 2011, a 32-year-old female presented to a gynecological clinic (Lensk, Russia) with a painful lesion on the right vulva arising after a trauma. Ultrasound examination revealed a round hypovascular mass, measuring 53×34×37 mm, in the soft tissues of the vulva. The patient underwent surgical excision of the mass. The conclusive diagnosis of the surgical specimen was of an organized hematoma.

A mass that was gradually increasingly painful was observed at the site of the excision at 7 months post-surgery. The patient was then referred to the Cancer Research Institute (Siberian Branch of the Russian Academy of Medical Sciences, Tomsk, Russia).

Gynecological examination revealed a tumor mass in the upper and middle thirds of the right labia majora, with involvement of the pubic area. The lesion consisted of multiple nodules with uneven density and well-defined borders, and was painful when palpated.

Histological analysis showed a spindle-like cell sarcoma [G2 (4)]. Examination of the tumor tissue revealed small monomeric cells with scant eosinophilic cytoplasm. Tumor cells formed straight or linear chains and exhibited anastomosis, forming an arcade pattern. The tumor structures were located in the prominent basophilic myxoid with a slightly vascular matrix. Hemorrhage and geographic necrosis were also evident in the tumor tissue.

The ultrasonographic findings revealed a dumbbell-shaped lesion measuring 27×70 mm, with a heterogeneous structure, sharp and smooth contour.

Spiral computed tomography scan revealed a well-defined multiple nodular lesion of soft-tissue density, actively accumulating the contrast, which was located in the soft tissues at the level of the symphysis pubis, on the right-hand side. The tumor extended to the right labia majora. Surrounding cellular tissue was thickened, but adjacent bone structures were unchanged. There was no evidence of lymph node involvement and no metastases were revealed.

No pathological changes were noted in the abdomen or chest.

**Figure 1.**
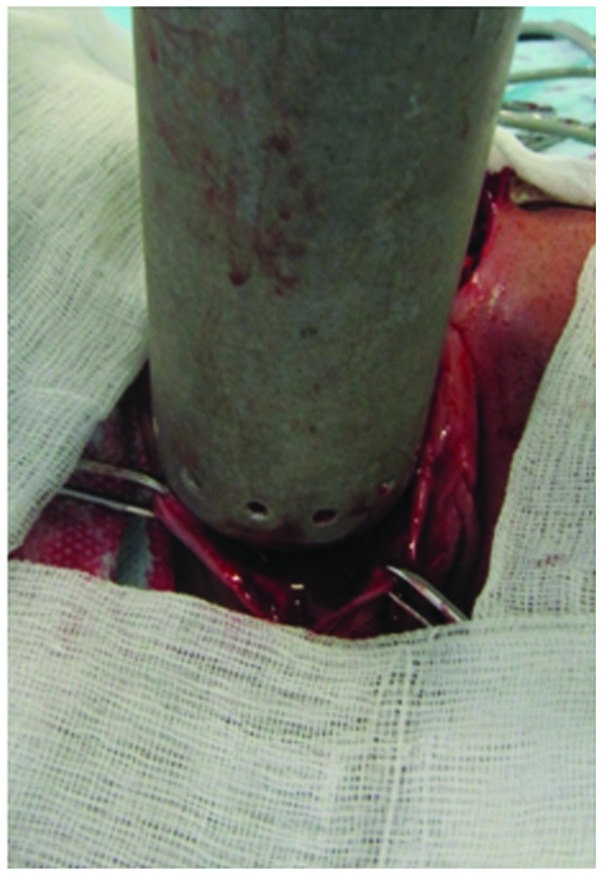
Surgical procedure. A collimator was positioned over the bed of the removed tumor for intraoperative radiation therapy.

The patient underwent wide excision of the tumor with reconstructive plastic surgery using local tissue flaps. The tumor was removed through a vertical incision. Cytological examination showed negative surgical margins. Intraoperative radiation therapy (IORT) at a single dose of 10 Gy was delivered to the bed of the removed tumor (Fig. 1). The wound was the closed layer by layer. There were no complications in the post-operative period.

**Figure 2.**
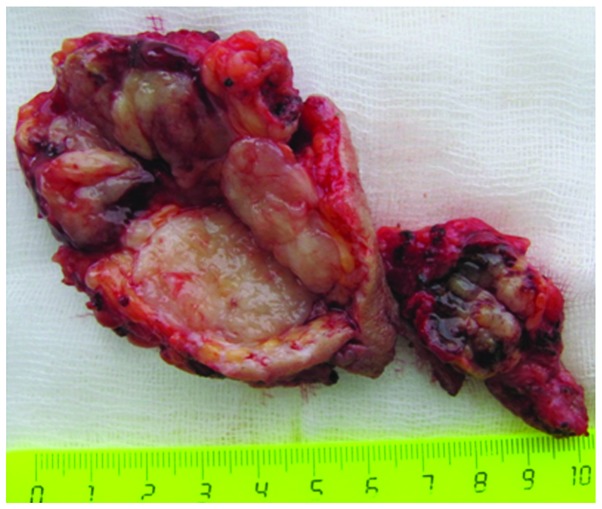
Macroscopic surgical specimen showing tumor nodules, measuring 5 cm at its widest, with a well-defined fibrotic capsule, and areas containing gelatinous material and hemorrhages.

Macroscopic imaging of the surgical specimen showed a tumor with multiple nodules, measuring 5 cm at its widest, which was mainly of elastic consistency, with a well-defined fibrotic capsule, and with areas containing gelatinous material and hemorrhages (Fig. 2).

**Figure 3.**
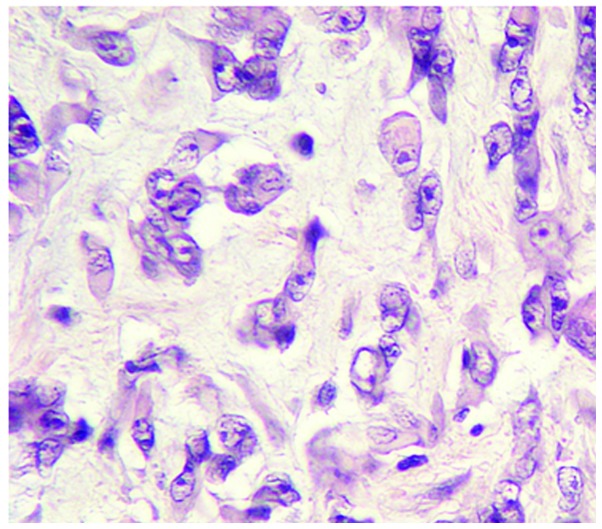
Polymorphic tumor cells with normochromic nuclei, large nucleoli and wide eosinophilic cytoplasm, forming short syncytial structures immersed in the abundant myxoid matrix (hematoxylin and eosin staining; magnification, x40).

**Figure 4.**
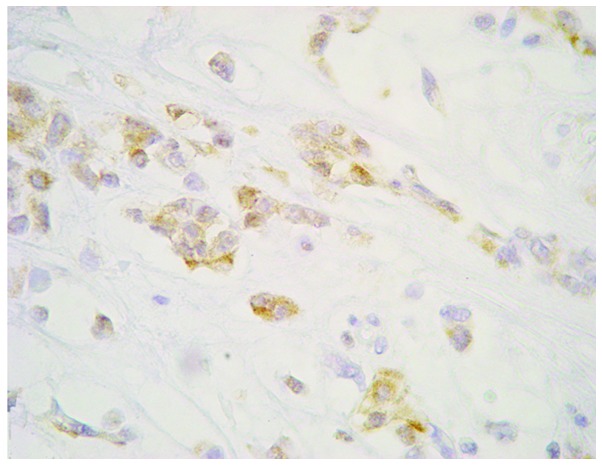
Diffuse cytoplasmic expression of neuron-specific enolase (magnification, х40).

A pathological study of this case revealed that the tumor was circumscribed by a pseudocapsule and consisted mainly of moderately polymorphic spindle-shaped cells and to a lesser extent, epithelioid cells, with swollen or prolate nuclei, vesicular chromatin, with one hypertrophic hyperchromic nucleolus and with bipolar amphophilic cytoplasmic processes. Cells formed syncytial, clustered and lacy structures immersed in the abundant myxoid matrix, with mild diffuse lymphoid infiltration. Fields of rhabdoid cells and foci of necrosis were observed within the tumor. A few atypical multinucleated giant cells were also present. Moderate mitotic activity with the presence of pathology forms was noted. The tumor tissue, mainly in peripheral areas, was divided into fields by fibrous bands with mild diffuse infiltration (Fig. 3). The majority of the tumor cells expressed neuron-specific enolase (monoclonal anti-human mouse antibody; clone BBS/NC/VI-H14; 1:600; Dako, Glostrup, Denmark) (Fig. 4). S-100 (monoclonal anti-human rabbit antibody; 1:600; Dako) was weakly expressed in the nuclei of certain cells. No expression was detected for pan-cytokeratin (monoclonal anti-human mouse antibody; 5/6/8/18; сlones 5D3 and LP34; 1:100; Leica Biosystems, Wetzlar, Germany), muscle actin (monoclonal anti-human mouse antibody; clone 1A4; 1:100; Dako), cluster of differentiation 34 (monoclonal anti-human mouse antibody; clone QBEnd 10; 1:100; Dako), desmin (monoclonal anti-human mouse antibody; clone DE-R-11; 1:100; Dako), MyoD1 (monoclonal anti-human mouse antibody; clone 5.8A; 1:140; Dako) and synaptophysin (monoclonal anti-human mouse antibody; clone SY38; 1:200; Dako). The proliferative activity of the tumor was high, with a Ki-67 of 26%.

The histological structure and immunophenotype of the tumor cells demonstrated an ESMC, G2 (4). Soft-tissue sarcoma of the vulva, stage Ib (T1bN0M0) was diagnosed.

The patient refused chemotherapy. A new lesion appeared in the right inguinal region 5 months after the surgery for recurrent vulvar cancer.

Magnetic resonance imaging showed heterogeneous enhancement of enlarged lymph nodes, measuring 37×25×34 mm, in the right inguinal region. Cytological examination revealed sarcoma cells. Due to disease progression, a bilateral inguinal-femoral lymph node dissection was performed. Metastatic involvement of ESMC in 1 of 13 lymph nodes was histologically confirmed. The patient subsequently received 40 Gy external beam radiation therapy to the inguinal region. The patient is currently alive with no evidence of disease progression at the 7 month follow-up.

## Discussion

ESMC of the vulva is an extremely rare malignant neoplasm. Only 4 clinical cases have been reported in the literature to date (38–41). The first clinical case was described in 1996, where the diagnosis of ESMC was established in a 40-year-old female with a tumor in the left labium majus. Following a wide excision of the vulvar tumor and inguinal lymphodissection, the patient was followed up for 40 months with no evidence of recurrence (39). The second clinical case of ESMC occurred in a 46-year-old female and was published in 2005 (40). In two other recent studies (2011), the patients were aged 24 and 66 years (38,41). In the 24-year-old female, the histological diagnosis was established only after total biopsy. The patient underwent vulvectomy with vulvoperitoneal reconstruction. Microscopic examination of the resected specimens revealed ESMC tumor nodules. However, no viable tumor cells were present at the surgical margin. The duration of recurrence-free follow-up was 2 years (41). The ESMC case of the 66-year-old patient was notable due to a prolonged postmenopausal period (20 years), large tumor size (8×12 сm) and combination treatment involving complete removal of the tumor and adjuvant radiation therapy. There was no evidence of recurrence after 1 year of follow-up (38).

There is little comparative data supporting the superiority of specific treatment regimens due to the low incidence of the ESMC.

In the present clinical case, ESMC was diagnosed too late for antitumor treatment to be effective. Improvement of optical imaging techniques, including histochemical methods, can contribute to the early diagnosis of ESMC of the vulva (9).

Due to the absence of a unified approach to the treatment of recurrent tumors, intraoperative radiation therapy to the bed of the removed tumor was selected in the present study, although there were have been no studies regarding the efficiency of IORT for vulvar sarcomas, including ESMC.

In spite of the obscure pathogenesis and absence of a pathogenetically proven therapeutic strategy for vulvar sarcomas, including ESMC of the vulva, the analysis of each clinical case has a high value and can contribute to the development of the optimal treatment policy.
